# Are socio-economic inequalities in diet and physical activity a matter of social distinction? A cross-sectional study

**DOI:** 10.1007/s00038-019-01268-3

**Published:** 2019-06-11

**Authors:** Joost Oude Groeniger, Carlijn B. M. Kamphuis, Johan P. Mackenbach, Mariëlle A. Beenackers, Frank J. van Lenthe

**Affiliations:** 1 Department of Public Health, Erasmus University Medical Centre, PO Box 2040, 3000 CA Rotterdam, The Netherlands; 2 Department of Interdisciplinary Sciences, Utrecht University, Utrecht, The Netherlands

**Keywords:** Socio-economic inequalities, Physical activity, Healthy diet, Social distinction

## Abstract

**Objectives:**

To explore whether ‘distinction’, a well-known mechanism that produces and reproduces social inequalities, can explain the socio-economic gradient in healthy diet and physical activity in contemporary obesogenic environments. If this is the case, we would expect a well-established indicator of distinction, ‘highbrow’ cultural participation, to be associated with a healthy diet and physical activity, while adjusting for education and income.

**Methods:**

Data from participants (25–75 years) of the 2014 wave of the Dutch GLOBE study (*N* = 2812) were used to analyse the association between ‘highbrow’ cultural participation (e.g. annual frequency of visits to museums, ballet, concerts, theatre) and sports participation, leisure-time walking and cycling, and fruit and vegetable intake, adjusted for education, income and other confounders.

**Results:**

Both highbrow cultural participation and healthy behaviours were more prevalent among high educational groups. Cultural participation was strongly associated with all health behaviours, even when adjusted for education and income.

**Conclusions:**

Our findings suggest that health behaviours, similar to highbrow cultural participation, are adopted as an expression of social distinction. This distinction mechanism may be an important determinant of health behaviour inequalities.

**Electronic supplementary material:**

The online version of this article (10.1007/s00038-019-01268-3) contains supplementary material, which is available to authorized users.

## Introduction

Diet and physical activity are strongly associated with socio-economic position. Those with higher levels of education or income are more likely to be physically active and to have a healthy diet, than those with lower levels of education or income (Beenackers et al. 2012; Giskes et al. 2010; Pampel et al. 2010). However, individual determinants directly related to educational or income level, such as knowledge and costs of a healthy lifestyle, can only partly explain health behaviour disparities (Ball et al. 2006; Kamphuis et al. 2008). Similarly, individual-level interventions aimed at improving health behaviours are often not effective, especially among lower socio-economic groups (Beauchamp et al. 2014). The mechanisms behind the association between socio-economic position and healthy dietary intake and physical activity are likely more complex, and require different perspectives.

While health-related behaviours are ultimately based on individual decisions, they are thought to be shaped and influenced by a lifelong socialization process (Abel 2008; Schori et al. 2014; Singh-Manoux and Marmot 2005). Socialization starts early in life and refers to the transmission of habits, social norms and preferences from within social groups to new members. Via socialization, habits, norms and preferences are internalized and converted into long-lasting dispositions, which play an important role in the establishment of lifestyles (Abel 2007; Bourdieu 1984; Cockerham 2005; Williams 1995). Since members from the same social classes often share the same experiences and environments (i.e. share a similar position in social space), there is also a high degree of affinity in lifestyles between them. These lifestyles become part of an identity and are used as a distinction mechanism to reflect differences between social groups (Abel 2007; Bourdieu 1984; Williams 1995).

With growing wealth, mass production and mass marketing, ‘conspicuous consumption’ and material goods are no longer the only marker of distinction (Currid-Halkett 2017; Van Eijck and Bargeman 2004). As compensation for the loss of social distinction on the basis of material possessions, one way for those from a higher social position to distinct one selves may be to adopt a healthy lifestyle (Currid-Halkett 2017; Mackenbach 2012). In contemporary obesogenic environments—environments that support, maintain and entice unhealthy behaviours and promote obesity in individuals or populations (Swinburn et al. 1999)—it requires more effort and restrain to act healthily than to act unhealthily. This makes a healthy lifestyle demanding and it provides higher-status groups with opportunities to distinguish themselves on the basis of health behaviours. For example, changes in the food system and food environment have resulted in an increasing availability, accessibility and visibility of high-energy and ultra-processed foods (Swinburn et al. 1999). Consequently, adopting healthy eating practices may be an increasingly relevant distinction practice in obesogenic environments. Research indeed suggests that effective resistance of unhealthy food environments depends on social class (Bissell et al. 2016), which may be related to differential dispositions across social groups. Similarly, with increasing opportunities for inactive and sedentary leisure-time activities (watching television, playing electronic games or other screen-related activities), being physically active in leisure time (e.g. walking, cycling or sports participation) diverges from the easy and default option of a sedentary lifestyle, which is likely encouraged by dispositions that attach more value to such leisure activities and which are likely more prevalent among higher-status groups.

The association between socio-economic position and physical activity and healthy dietary intake may thus reflect a broader spectrum of cultural preferences and tastes related to the social position of people (Pampel 2012), which is used as a means for social distinction. This mechanism may lead to more healthy behaviours in high socio-economic groups, both consciously (thereby fulfilling a need to distinguish one selves’ from other social groups) and unconsciously (as an unconscious expression of internalized tastes, preferences and attitudes as developed through socialization processes within one’s specific sociocultural environment).

To test whether healthy dietary intake and physical activity also result from practices and attitudes used for social distinction, we need to study whether these health behaviours are associated with a well-known indicator of social distinction. And preferably an indicator that is not likely to be associated with the health behaviours in a causal way. Building upon the work of Bourdieu, ‘highbrow’ cultural participation is probably the best available indicator of social distinction by high-status groups. Cultural participation reflects a disposition for ‘highbrow’ cultural practices (e.g. arts) (Bourdieu 1984; Bourdieu et al. 1991; Lamont and Lareau 1988; Nagel and Ganzeboom 2015) and is most often used to measure one’s embodied cultural capital (Kamphuis et al. 2015a). A disposition for these types of cultural practices is not easily acquired, since the appreciation and comprehension of cultural activities have to be learned and require time and effort. Moreover, the process of becoming familiar with cultural practices can only be learned from those who possess this cultural familiarity themselves. This provides high-status groups, who more often possess these familiarities and pass them on from one generation to the next (Kraaykamp and van Eijck 2010), with the resources to use cultural participation as an instrument of social distinction (Bourdieu 1984; Bourdieu et al. 1991; Nagel and Ganzeboom 2015).

Participation in ‘highbrow’ cultural activities thus reflects a socialization process in sociocultural environments with a preference for ‘sophisticated’ lifestyles and a tendency for social distinction. We hypothesize that—due to the same underlying socialization mechanisms—both cultural participation and healthy behaviours are a way of social distinction. To test this hypothesis, we examined whether cultural participation is associated with sports participation, walking and cycling in leisure time, and fruit and vegetable consumption, independent of education, income and a set of potential confounders.

## Methods

Data were collected by means of a large-scale postal survey in 2014, administered as the fifth wave of data collection for the longitudinal Dutch GLOBE (Dutch acronym for Health and Living Conditions of the Population of Eindhoven and surroundings) study (response = 45.5%). All data were self-reported and collected between October and December 2014. The cross-sectional sample of the data collection formed a stratified sample of the 25–75-year-old population in the city of Eindhoven and was used in the analyses (*N* = 2812). More detailed information on the objectives, study design and data collection of the Dutch GLOBE study can be found elsewhere (Van Lenthe et al. 2014). The use of personal data in the GLOBE study is in compliance with the Dutch Personal Data Protection Act and the Municipal Database Act, and has been registered with the Dutch Data Protection Authority (Number 1248943).

### Exposures

Cultural participation was measured by asking respondents how often they visit art museums, historical museums, opera or ballet, classical concerts, theatre and architecture (the most commonly used cultural activities to measure ‘highbrow’ embodied cultural capital (Kamphuis et al. 2015a). Answer categories were: 0—never, 1—once per year and 2—more than once per year.

Principal component analysis (PCA) showed that all cultural activities loaded clearly on one latent factor (Eigenvalue = 2.97) and subsequent reliability analysis showed that it was possible to construct a reliable scale from the cultural activities (Cronbach’s alpha = 0.786). Consequently, all items were summed to form a scale ranging from 0 to 14. This scale was subsequently divided into quintiles, so that one higher quintile corresponds to two additional ‘highbrow’ cultural visits.

Highest attained educational level was classified according to the International Standard Classification of Education (ISCED): 1—primary education (ISCED 0–1), 2—lower secondary education (ISCED 2), 3—upper secondary education (ISCED 3–4) and 4—tertiary education (ISCED 5–7).

Household equivalent income was measured as the level of household income per month divided by the square root of the number of people living from this income. This method is often used and takes differences in needs for households of different sizes into account (OECD 2008). Income was subsequently divided into five categories: 1—< €1000/month, 2—€1000–€1500/month, 3—€1500–€2000/month, 4—€2000–2500/month and 5— > €2500/month. Sensitivity analyses using non-equivalized income categories (i.e. €0–1200, €1200–1800, €1800–2600, €2600–4000, > €4000) showed similar results (results not shown).

### Outcomes

Sports participation was measured using the validated Short QUestionnaire to ASsess Health-enhancing physical activity (SQUASH) (Wendel-Vos et al. 2003). Participants were asked to write down up to four sports they had participated in on a weekly basis in the last month. For each sport, they reported frequency (days per week), duration (hours and minutes per day) and intensity (light, moderate, intense). Self-reported intensity and activity-specific intensity metabolic equivalents (METs) were used to calculate the numbers of days participants were active for at least 30 min at moderate or vigorous intensity (moderate intensity = 4–6 MET for 18–55 years and 3–5 MET for 55 + years). Because of skewness, the variable was dichotomized into not doing any sports for 30 min or more at moderate intensity, versus sports participation at least once per week for 30 min or more at moderate intensity.

Walking or cycling in leisure time was also measured with the SQUASH. Participants were asked how often they walked and cycled in their leisure time. For both activities, frequency (days per week), duration (hours and minutes per day) and intensity (light, moderate and intense) were reported. Subsequently, we calculated how many days per week the participant walked or cycled for at least 30 min at moderate intensity. This cut-off was chosen because 30 min or more of walking or cycling implies that the participant made the effort to be physically active rather than just a means of local transportation (e.g. grocery shopping). Because of skewness, the variable was dichotomized into not walking or cycling in leisure time for 30 min or more at moderate intensity versus walking or cycling at least once per week for 30 min or more at moderate intensity.

Fruit and vegetable consumption was measured with a food frequency questionnaire (Bogers et al. 2004). Participants were asked how many times, on a weekly basis, they had consumed fruit and vegetables in the previous month. Subsequently they were asked to indicate how many portions (pieces of fruit and serving spoons [= 50 g] of vegetables) they ate on a typical occasion. For ease of interpretation, we calculated binary measures that indicated whether or not participants meet fruit and vegetable intake recommendations. The consumption of two pieces of fruit each day was coded as meeting recommend fruit intake. The consumption of at least 200 grams of vegetables each day was coded as meeting recommended vegetable intake.

### Confounders

Potential confounders included were sex (male and female), age (in 10-year age groups), living together with a partner (yes, no), country of birth (the Netherlands, other), having children living at home (yes, no), employment status (full-time employed, part-time employed, unemployed, retired, homemaker, other), social participation (at least once per week, several times per month, once per month, several times per year, once per year or less) in several associations and organizations (neighbourhood association, political organization, social club, religious organization, work-related organization [e.g. labour union], sport club or volunteer organization), mental health [five-item version of the Mental Health Inventory [MHI-5] (Berwick et al. 1991)] and self-rated health (excellent, very good, good, fair, poor).

### Statistical analysis

Multivariable regression analyses were used to analyse the association between cultural participation and the health-related behaviours. First, regression models were calculated to estimate the association between cultural participation and outcomes, adjusted for potential confounders. Second, education and income were added to the model to estimate the independent associations between cultural participation, education and income and the outcomes. We used generalized linear regression models with log link function and robust variance in order to derive prevalence ratios (PR) and 95% confidence intervals (CI).

Missing data were handled via multiple imputations. Twenty imputed datasets were created. Information from all variables described in the methods and several other variables is used in the imputation model. Respondents with missing data on an outcome variable were left out of the analyses concerning that outcome variable (9.7% missing on sports participation, 3.8% missing on walking and cycling in leisure time, 2.0% missing on recommended fruit intake and 2.2% missing on recommended vegetable intake). The analyses were weighted by respondent-level sample weights to account for the sampling strategy within the GLOBE study. All analyses were performed using Stata, version 15 (StataCorp LP, College Station, Texas).

## Results

The mean age of the sample was 48.9 (standard deviation 15.6) and 55% was female. Sixty-one per cent of the respondents participated at least once per week in sports for 30 min or more at moderate or vigorous intensity, 65% walked or cycled at least once per week for 30 min in leisure time, 77% met vegetable intake recommendations and 66% met fruit intake recommendations (Table 1).

**Table 1:** Descriptive statistics of the participants (Netherlands 2014, GLOBE: Dutch acronym of Health and Living Conditions of the Population of Eindhoven and surroundings)

Variables	Percentage
Gender	
Men	44.8%
Women	55.2%
Age groups	
25–34 years	26.2%
35–44 years	19.6%
45–54 years	14.3%
55–64 years	16.1%
65–75 years	23.8%
Living together with a partner	
Yes	73.8%
No	26.2%
Country of birth	
The Netherlands	88.6%
Else	11.4%
Children living at home	
No	65.5%
Yes	34.5%
Employment status	
Full-time employed	37.6%
Part-time employed	23.7%
Unemployed	8.1%
Retired	22.5%
Homemaker	4.8%
Other	3.3%
Social participation	
No participation	36.3%
Once per year or less	7.4%
Couple times per year	9.1%
Once per month	4.7%
Couple times per month	10.0%
At least once per week	32.7%
Self-rated health	
Excellent	14.0%
Very good	29.5%
Good	42.3%
Fair	12.5%
Poor	1.8%
Mental health	
MHI-5 (mean ± standard deviation)	4.6 ± 0.8
Cultural participation	
1 lowest (0–1 visits per year)	22.2%
2 (at least 2 visits per year)	23.8%
3 (at least 4 visits per year)	22.1%
4 (at least 6 visits per year)	17.3%
5 highest (at least 8 visits per year)	14.6%
Educational level	
Primary	5.7%
Lower secondary	22.1%
Upper secondary	24.9%
Tertiary	47.3%
Household equivalent income	
< €1000/month	14.3%
€1000–€1500/month	20.7%
€1500–€2000/month	24.8%
€2000–€2500/month	28.1%
> €2500/month	12.1%
Health behaviours	
Sports participation	61.4%
Walking or cycling in leisure time	65.4%
Recommended vegetable intake	76.5%
Recommended fruit intake	66.3%

Descriptive statistics calculated on non-imputed data

Those with a higher level of education or income had higher levels of cultural participation (Online Resource Figure S1). Spearman correlations were moderate: 0.41 between cultural participation and education, 0.37 between cultural participation and income and 0.41 between education and income. Those with a higher level of education were more likely to participate in sports, walk or cycle in leisure time and adhere to vegetable intake recommendations (Table 2). Those with a higher level of income were more likely to participate in sports and to meet fruit and vegetable intake recommendations.

**Table 2:** Prevalence ratios between educational level and health behaviours and income and health behaviours (Netherlands 2014, GLOBE: Dutch acronym of Health and Living Conditions of the Population of Eindhoven and surroundings)

	Sports participation		Walking or cycling in leisure time		Recommended vegetable intake		Recommended fruit intake	
	PR (95% CI)	*p*	PR (95% CI)	*p*	PR (95% CI)	*p*	PR (95% CI)	*p*
Educational level								
Primary	1		1		1		1	
Lower secondary	1.90 (1.36–2.65)	< 0.001	1.09 (0.96–1.24)	0.186	1.44 (0.88–2.36)	0.146	0.87 (0.66–1.15)	0.336
Upper secondary	2.02 (1.45–2.80)	< 0.001	1.09 (0.95–1.25)	0.197	1.39 (0.85–2.27)	0.194	0.86 (0.64–1.14)	0.291
Tertiary	2.24 (1.61–3.10)	< 0.001	1.16 (1.02–1.33)	0.026	2.12 (1.31–3.43)	0.002	1.03 (0.78–1.36)	0.836
Household equivalent income								
< €1000/month	1		1		1		1	
€1000–€1500/month	1.26 (1.07–1.47)	0.005	0.97 (0.86–1.08)	0.549	1.11 (0.81–1.52)	0.499	1.21 (0.95–1.55)	0.126
€1500–€2000/month	1.36 (1.16–1.60)	< 0.001	1.06 (0.94–1.18)	0.345	1.25 (0.91–1.73)	0.174	1.31 (1.02–1.69)	0.033
€2000–€2500/month	1.34 (1.14–1.56)	< 0.001	1.04 (0.93–1.16)	0.533	1.40 (1.03–1.91)	0.032	1.37 (1.07–1.76)	0.012
> €2500/month	1.47 (1.24–1.74)	< 0.001	1.09 (0.96–1.24)	0.184	1.51 (1.07–2.12)	0.019	1.40 (1.06–1.84)	0.018

All models were adjusted for confounders: sex, age, living together with a partner, country of birth, children living at home, employment status, social participation, mental health and self-rated health

*PR* prevalence ratio, *CI* confidence interval

Cultural participation was associated with all four outcome variables in regression models that did not adjust for education and income (crude models in Tables 3, 4, 5, 6). These associations showed a clear gradient; those with higher levels of cultural participation were more likely to participate in sports, walk or cycle in leisure time and were more likely to meet fruit and vegetable intake recommendations.

**Table 3:** Prevalence ratios of the association between cultural participation, educational level and income and sports participation (Netherlands 2014, GLOBE: Dutch acronym of Health and Living Conditions of the Population of Eindhoven and surroundings); *N* = 2583

Variables	Categories	Crude model				Education and income				Adjusted model			
		PR	95% CI		*p*	PR	95% CI		*p*	PR	95% CI		*p*
Cultural participation (quintiles)	1 lowest	1								1			
	2	1.25	1.10	1.42	< 0.001					1.16	1.02	1.31	0.02
	3	1.34	1.18	1.52	< 0.001					1.22	1.07	1.38	0.002
	4	1.38	1.22	1.56	< 0.001					1.24	1.09	1.40	0.001
	5 highest	1.37	1.20	1.56	< 0.001					1.21	1.06	1.39	0.005
Educational level	Primary					1							
	Lower secondary					1.81	1.30	2.53	< 0.001	1.72	1.23	2.40	0.001
	Upper secondary					1.90	1.37	2.65	< 0.001	1.77	1.26	2.47	0.001
	Tertiary					2.08	1.50	2.90	< 0.001	1.88	1.34	2.63	< 0.001
Household equivalent income	< €1000					1				1			
	€1000–€1500					1.20	1.02	1.41	0.024	1.18	1.01	1.38	0.039
	€1500–€2000					1.27	1.08	1.48	0.004	1.23	1.05	1.44	0.012
	€2000–€2500					1.21	1.03	1.42	0.017	1.17	1.00	1.36	0.056
	> €2500					1.31	1.10	1.56	0.002	1.26	1.06	1.50	0.009

All models were adjusted for confounders: sex, age, living together with a partner, country of birth, children living at home, employment status, social participation, mental health and self-rated health

*PR* prevalence ratio, *CI* confidence interval

**Table 4:** Prevalence ratios of the association between cultural participation, educational level and income and walking or cycling in leisure time (Netherlands 2014, GLOBE: Dutch acronym of Health and Living Conditions of the Population of Eindhoven and surroundings); *N* = 2705

Variables	Categories	Crude model				Education and income				Adjusted model			
		PR	95% CI		*p*	PR	95% CI		*p*	PR	95% CI		*p*
Cultural participation (quintiles)	1 lowest	1								1			
	2	1.15	1.05	1.27	0.004					1.15	1.05	1.27	0.004
	3	1.23	1.12	1.35	< 0.001					1.23	1.11	1.36	< 0.001
	4	1.23	1.12	1.36	< 0.001					1.24	1.11	1.37	< 0.001
	5 highest	1.34	1.22	1.46	< 0.001					1.34	1.21	1.49	< 0.001
Educational level	Primary					1				1			
	Lower secondary					1.09	0.95	1.24	0.223	1.02	0.89	1.17	0.751
	Upper secondary					1.08	0.94	1.25	0.255	0.99	0.86	1.14	0.898
	Tertiary					1.15	1.00	1.32	0.058	1.01	0.87	1.17	0.907
Household equivalent income	< €1000					1				1			
	€1000–€1500					0.96	0.85	1.07	0.427	0.94	0.84	1.05	0.258
	€1500–€2000					1.04	0.92	1.16	0.546	1.00	0.89	1.12	0.986
	€2000–€2500					1.00	0.89	1.13	0.943	0.95	0.84	1.07	0.379
	> €2500					1.05	0.92	1.20	0.488	0.99	0.87	1.14	0.900

All models were adjusted for confounders: sex, age, living together with a partner, country of birth, children living at home, employment status, social participation, mental health and self-rated health

*PR* prevalence ratio, *CI* confidence interval

**Table 5:** Prevalence ratios of the association between cultural participation, educational level and income and recommended vegetable intake (Netherlands 2014, GLOBE: Dutch acronym of Health and Living Conditions of the Population of Eindhoven and surroundings); *N* = 2749

Variables	Categories	Crude model				Education and income				Adjusted model			
		PR	95% CI		*p*	PR	95% CI		*p*	PR	95% CI		*p*
Cultural participation (quintiles)	1 lowest	1								1			
	2	1.01	0.77	1.33	0.946					0.93	0.70	1.24	0.642
	3	1.39	1.07	1.8	0.013					1.23	0.94	1.62	0.130
	4	1.63	1.26	2.13	< 0.001					1.40	1.05	1.86	0.021
	5 highest	1.88	1.44	2.45	< 0.001					1.57	1.17	2.10	0.003
Educational level	Primary					1				1			
	Lower secondary					1.39	0.85	2.29	0.193	1.32	0.79	2.22	0.289
	Upper secondary					1.33	0.81	2.18	0.267	1.20	0.71	2.01	0.497
	Tertiary					1.98	1.21	3.23	0.007	1.62	0.96	2.74	0.069
Household equivalent income	< €1000					1				1			
	€1000–€1500					1.06	0.78	1.45	0.719	1.03	0.75	1.41	0.859
	€1500–€2000					1.14	0.82	1.57	0.434	1.08	0.78	1.50	0.640
	€2000–€2500					1.19	0.86	1.63	0.291	1.08	0.79	1.49	0.626
	> €2500					1.22	0.85	1.74	0.284	1.09	0.76	1.56	0.638

All models were adjusted for confounders: sex, age, living together with a partner, country of birth, children living at home, employment status, social participation, mental health and self-rated health

*PR* prevalence ratio, *CI* confidence interval

**Table 6:** Prevalence ratios of the association between cultural participation, educational level and income and recommended fruit intake (Netherlands 2014, GLOBE: Dutch acronym of Health and Living Conditions of the Population of Eindhoven and surroundings); *N* = 2757

Variables	Categories	Crude model				Education and income				Adjusted model			
		PR	95% CI		*p*	PR	95% CI		*p*	PR	95% CI		*p*
Cultural participation (quintiles)	1 lowest	1								1			
	2	1.05	0.85	1.28	0.667					1.06	0.86	1.31	0.578
	3	1.17	0.96	1.43	0.114					1.18	0.96	1.47	0.121
	4	1.31	1.07	1.61	0.008					1.31	1.05	1.64	0.016
	5 highest	1.57	1.3	1.91	< 0.001					1.58	1.27	1.96	< 0.001
Educational level	Primary					1				1			
	Lower secondary					0.81	0.61	1.08	0.148	0.76	0.57	1.02	0.063
	Upper secondary					0.79	0.59	1.06	0.113	0.70	0.52	0.95	0.022
	Tertiary					0.93	0.69	1.24	0.610	0.76	0.56	1.04	0.088
Household equivalent income	< €1000					1				1			
	€1000–€1500					1.24	0.96	1.59	0.100	1.21	0.94	1.57	0.134
	€1500–€2000					1.33	1.03	1.72	0.030	1.28	0.98	1.67	0.067
	€2000–€2500					1.36	1.05	1.77	0.022	1.25	0.95	1.63	0.105
	> €2500					1.36	1.01	1.82	0.041	1.24	0.92	1.67	0.154

All models were adjusted for confounders: sex, age, living together with a partner, country of birth, children living at home, employment status, social participation, mental health and self-rated health

*PR* prevalence ratio, *CI* confidence interval

When education and income were included in the regression models, the estimated associations between cultural participation and the health-related behaviours reduced moderately, but remained strong for all outcomes (adjusted models in Tables 3, 4, 5, 6). Compared to participants in the lowest quintile of cultural participation, those in the highest quintile were more likely to participate in sports (PR 1.21, 95% CI 1.06; 1.39), walk and cycle in leisure time (PR 1.34, 95% CI 1.21; 1.49), meet vegetable intake recommendations (PR 1.57, 95% CI 1.17; 2.10) and meet fruit intake recommendations (PR 1.58, 95% CI 1.27; 1.96). Whereas cultural participation was statistically significantly associated with all outcomes, educational level and household equivalent income were only statistically significantly (at the 5% level) associated with sports participation (tertiary versus primary: PR 1.88, 95% CI 1.34; 2.63 and < €1000/month versus > €2500/month: PR 1.26, 95% CI 1.06; 1.50).

Stratified analyses showed that the association between cultural participation and recommended fruit and vegetable intake was slightly stronger among women than among men, and whereas the association between cultural participation and sport participation was stronger among higher age groups, the association between cultural participation and walking or cycling and recommended vegetable intake was stronger among lower age groups (Online Resource Tables S11–S15).

## Discussion

### Summary of main findings

In the present sample of Dutch adults, those who had a higher level of cultural participation were more likely to participate in sports, to walk and cycle in leisure time and to meet fruit and vegetable intake recommendations. These associations showed a clear gradient, even when adjusted for education and income and a range of potential confounders.

### Interpretation of the findings

The strong association between cultural participation and the health-related behaviours presumably does not suggest that engaging in cultural activities will directly motivate individuals to engage in more healthy behaviour. Rather, it suggests that socialization in high-status environments results in tastes, preferences and attitudes that induces a tendency to consume more highbrow cultural activities as well as a tendency to eat more healthily and to be more physically active during leisure time. This argument is perhaps best exemplified by the results pertaining to walking and cycling in leisure time. Our study found that walking and cycling were independently associated with cultural participation, but not with education and income. Since these activities do not require many financial resources, or specific knowledge or skills, there is no reason for a strong causal relationship with income or education. Going for a walk or a bicycle ride in leisure time does, however, require a certain appreciation for spending leisure time carrying out these activities. This appreciation is likely developed by the same socialization processes in which enjoyment for cultural activities is attained. Walking and cycling in leisure time can thus be viewed as an expressions of taste which is also associated with being culturally active and which allows higher-status groups to distinguish themselves from others.

The association between cultural participation and sports participation similarly suggests that sports are an important part of lifestyles and act as a marker of distinction. Indeed, research has shown that different social groups participate in different types of sport (Bourdieu 1984; Kraaykamp et al. 2013; Pampel et al. 2010; Stempel 2005; Wilson 2002). For the purposes of our study, we did not specifically examine the distribution of different types of sports, but rather we examined whether the socio-economic gradient in sports involvement reflects cultural differences between socio-economic groups. Similarly, Christensen and Carpiano (2014) also found that those with higher levels of cultural capital were more likely to be involved in sports. However, in line with studies that have observed social class differentiation in types of sports (Bourdieu 1984; Scheerder et al. 2002; Stempel 2005; Wilson 2002), restricting our analysis to ‘high-status’ sports (e.g. golf, tennis, sailing) also resulted in a strong association between cultural participation and sports involvement (Online Resource Table S8). Moreover, associations with cultural participation were not observed when we restricted the analysis to sports that were not defined as ‘high-status’ sports (Online Resource Table S9). In addition to a mechanism of distinction, the association between educational and income level and sports participation suggests that other determinants related to education or income, such as financial resources or social capital (e.g. social support), are also important for sports participation. Again, this is also in line with existing research, since economic capital (i.e. income-related resources) has been found to be associated with a higher probability of being involved in sports, independent of other capital measures (Christensen and Carpiano 2014; Wilson 2002).

The strong association between cultural participation and meeting fruit and vegetable intake recommendations again likely represents the tastes and attitudes of different social groups (Fismen et al. 2012). Eating patterns and food preferences are subject to socialization processes and are internalized and converted into dispositions. Several studies have shown that high cultural capital is associated with healthier food consumption (Fismen et al. 2012; Kamphuis et al. 2015a; Kraaykamp 2002). For example, Van Otterloo and Ogtrop (1989) found that food choices among Dutch mothers from lower social classes could be characterized by a regime of ‘much, fat and sweet’, whereas mothers from higher social classes paid more attention to the healthiness of their nutritional patterns. Kraaykamp (2002) also observed taste differences between social groups in Dutch society and showed that cultural capital provides higher-status groups with resources to adopt a more exclusive consumption pattern. Kamphuis et al. (Kamphuis et al. 2015b) have recently showed that higher educational groups in the Netherlands were more concerned with healthiness than lower education groups. These results are in line with observations by Bourdieu, who suggested that lower-status groups are more inclined to eat traditional, heavier and cheaper foods, whereas higher-status group has a preference for more delicate, refined and healthy foods (Bourdieu 1984). Although differences in food consumption between social groups are much more encompassing, we restricted this study to fruit and vegetable consumption because differences in fruit and vegetable consumption are the most profound contributors to socio-economic inequalities in healthy dietary intake (Giskes et al. 2010). To test the hypothesis related to food preferences more directly we have previously examined the association between cultural participation and so-called superfoods (i.e. spelt, quinoa, goji berry, chia seeds and wheatgrass), the consumption of which can, in our view, be seen as a contemporary expression of social distinction. These results also showed a strong association between cultural participation and the consumption of superfoods, net of education and income (Oude Groeniger et al. 2017), supporting our interpretation that differences in healthy food consumption can be seen as an expression of distinction.

### Limitations of the study

This study is not without limitations. First, we have interpreted the strong association between cultural participation and health-related behaviour as evidence for a social distinction mechanism. We feel confident about this interpretation since ‘highbrow’ cultural participation is a well-known indicator of embodied cultural capital and a clear marker of distinction. Moreover, cultural participation is not likely associated with health behaviour in a causal way (i.e. the mere act of cultural participation, such as visiting museums or classical concerts, is not likely to cause a strong increase in fruit consumption or leisure-time walking). However, it could be argued that other determinants may be responsible for the association between cultural participation and health-related behaviours. One is the amount of leisure time available, as more leisure time may clearly increase one’s available time to spend on both cultural participation and health-related behaviours, like walking, cycling and sports participation. We had no information on respondents’ amount of leisure time, but we tried to adjust for this factor by controlling all analyses for two important time restriction variables: employment status and whether the respondent had children living at home (Kraaykamp et al. 2008). Cognitive ability may also have confounded our results. It has been argued that cultural participation is higher among people with greater information processing capacities, because they seek more complex cultural activities to fulfil their needs (Notten et al. 2015), and cognitive ability may also result in a more healthy lifestyle. However, by conditioning on educational level we have likely accounted for a substantial part of this confounding effect. Further, cultural participation may reflect an active lifestyle rather than high cultural tastes, and this active lifestyle may also result in more sports participation and more leisure-time physical activity. Although we adjusted for social participation in several associations and organizations, there may be other determinants that affect both cultural participation and health-related behaviours that were not controlled for in our analyses.

Second, we only used a limited number of cultural activities to represent cultural participation, and cultural participation only measures one facet of the sociocultural environment of the participants. For our purposes, trying to reflect a social distinction mechanism, ‘highbrow’ cultural participation is an important and strong indicator (Bourdieu 1984; Bourdieu et al. 1991; Nagel and Ganzeboom 2015; Notten et al. 2015). Moreover, we have used the most commonly applied ‘highbrow’ cultural activities in our study (Kamphuis et al. 2015a). Notwithstanding, there are likely more relevant cultural activities and social distinction instruments (e.g. possession of arts at home, music preferences) that we have not captured in our analyses.

Third, we used self-reported information about the health behaviours. If higher socio-economic groups indeed value a healthy lifestyle more than people from lower socio-economic groups, as we hypothesized, they may be more inclined to overestimate their healthy behaviours or underreport their weight. However, the use of the SQUASH for physical activity has been shown to be reliable and valid (Wendel-Vos et al. 2003) and the use of a food frequency questionnaire was considered suitable for ranking individuals and establishing relative intake (Bogers et al. 2004).

Fourth, the use of cross-sectional data only offers a snapshot of the social and cultural processes through which dietary habits and physical activity are formed and limits the ability to make any causal interpretations.

### Implications and future research

The proposed mechanism of social distinction reflects a long-lasting process of socialization that constrains and guides behaviours throughout the life course. If behaviours are not consistent with the habits and preferences of one’s sociocultural environment, it is unlikely that they will be materialized or maintained. This may contribute to the poor effectiveness of many interventions aimed at improving health behaviours, especially among lower socio-economic groups (Beauchamp et al. 2014). To improve their effectiveness, interventions should be better aligned to what is considered meaningful and practicable in different sociocultural environments (Delormier et al. 2009). In addition, because socialization plays a strong role in the adoption of health-related behaviours, interventions focusing on young children and their parents may be most successful, as incorporating healthy habits then becomes part of the socialization process.

More research, however, is needed for more in-depth examinations of these mechanisms and the potential to intervene on them. The hypothesis that social distinction induces a healthy lifestyle in higher socio-economic groups should also be tested in a more direct manner. Since the process of distinction can be implicit, this will require more sophisticated (experimental) methods combined with more qualitative studies. One method that may be able to measure a tendency for social distinction more directly—even if this tendency is unconscious—is the use of Implicit Association Tests. Implicit Association Tests are specifically designed to include unconscious attitudes of subjects (Greenwald et al. 1998), which could be very useful when assessing a tendency for social distinction. Techniques that are used in research on social comparison may also be helpful in this line of inquiry. For instance, prototype perceptions (i.e. social images of typical persons engaging in certain behaviours) have been used to study health risk behaviours (Gibbons and Gerrard 1995). Similar techniques may also be effective in assessing social distinction tendencies. In addition, more research is needed to disentangle how these mechanisms may differ across gender and age groups.

### Conclusions

This study suggests that healthy dietary patterns and physical activity are adopted as an expression of social distinction, similar to cultural participation. This mechanism may be an important determinant of health behaviour inequalities and should not be neglected when planning interventions to reduce these inequalities. More research is needed for in-depth examinations of these mechanisms and its consequences for contemporary health inequalities.

## Electronic supplementary material

Below is the link to the electronic supplementary material.

Supplementary material 1 (DOCX 184 kb)
