# Supplementary material for: Are socio-economic inequalities in diet and physical activity a matter of social distinction? A cross-sectional study
Source: Int J Public Health. 2019 Jun 11;64(7):1037–47. doi: 10.1007/s00038-019-01268-3 (PMC6677869; doi:10.1007/s00038-019-01268-3)
Supplement: Supplementary file 1 — Supplementary material 1 (DOCX 184 kb) [file 38_2019_1268_MOESM1_ESM.docx]

**Electronic Supplementary Material**

**Detailed information on cultural participation**

Supplementary Table S1. Frequency of visiting cultural activities (Netherlands 2014, GLOBE: Dutch acronym of Health and Living Conditions of the Population of Eindhoven and surroundings).

|  | Never | Once per year | >Once per year |
| --- | --- | --- | --- |
| Art museums | 53.9% | 30.4% | 15.7% |
| Historical museums | 54.9% | 37.1% | 8.1% |
| Opera | 85.5% | 11.5% | 3.0% |
| Classical concerts | 78.2% | 14.8% | 7.0% |
| Theatre | 43.5% | 37.4% | 19.0% |
| Architecture | 37.1% | 30.6% | 32.3% |

Supplementary Table S2. Frequency of visiting cultural activities by gender (Netherlands 2014, GLOBE: Dutch acronym of Health and Living Conditions of the Population of Eindhoven and surroundings).

|  | Never | Once per year | >Once per year |
| --- | --- | --- | --- |
| **Men** |  |  |  |
| Art museums | 53.4% | 31.1% | 15.5% |
| Historical museums | 51.3% | 39.5% | 9.1% |
| Opera | 87.6% | 9.9% | 2.5% |
| Classical concerts | 78.8% | 14.9% | 6.3% |
| Theatre | 46.8% | 36.8% | 16.3% |
| Architecture | 35.6% | 29.6% | 34.8% |
| **Women** |  |  |  |
| Art museums | 54.3% | 29.8% | 15.9% |
| Historical museums | 57.7% | 35.1% | 7.2% |
| Opera | 83.8% | 12.8% | 3.5% |
| Classical concerts | 77.7% | 14.7% | 7.6% |
| Theatre | 40.8% | 37.9% | 21.3% |
| Architecture | 38.3% | 31.4% | 30.3% |

Supplementary Table S3. Frequency of visiting cultural activities by age group (Netherlands 2014, GLOBE: Dutch acronym of Health and Living Conditions of the Population of Eindhoven and surroundings).

|  | Never | Once per year | >Once per year |
| --- | --- | --- | --- |
| **Age 25-34** |  |  |  |
| Art museums | 53.1% | 36.8% | 10.0% |
| Historical museums | 55.5% | 39.1% | 5.4% |
| Opera | 87.4% | 10.9% | 1.7% |
| Classical concerts | 86.5% | 10.8% | 2.8% |
| Theatre | 34.6% | 49.0% | 16.3% |
| Architecture | 35.9% | 32.7% | 31.3% |
| **Age 35-44** |  |  |  |
| Art museums | 56.9% | 30.9% | 12.2% |
| Historical museums | 54.2% | 37.1% | 8.7% |
| Opera | 88.3% | 9.9% | 1.9% |
| Classical concerts | 82.1% | 14.8% | 3.2% |
| Theatre | 37.7% | 41.7% | 20.6% |
| Architecture | 37.4% | 34.9% | 27.7% |
| **Age 45-55** |  |  |  |
| Art museums | 56.9% | 28.0% | 15.1% |
| Historical museums | 60.3% | 32.9% | 6.8% |
| Opera | 86.2% | 11.1% | 2.8% |
| Classical concerts | 76.9% | 17.3% | 5.8% |
| Theatre | 45.5% | 34.3% | 20.2% |
| Architecture | 37.7% | 30.7% | 31.7% |
| **Age 55-64** |  |  |  |
| Art museums | 49.2% | 30.9% | 19.9% |
| Historical museums | 51.2% | 40.3% | 8.5% |
| Opera | 83.5% | 12.1% | 4.4% |
| Classical concerts | 72.7% | 17.0% | 10.3% |
| Theatre | 47.3% | 29.1% | 23.6% |
| Architecture | 35.0% | 25.9% | 39.1% |
| **Age 65-74** |  |  |  |
| Art museums | 53.5% | 24.1% | 22.4% |
| Historical museums | 53.9% | 35.3% | 10.8% |
| Opera | 82.0% | 13.2% | 4.8% |
| Classical concerts | 70.4% | 16.2% | 13.4% |
| Theatre | 54.8% | 28.3% | 16.9% |
| Architecture | 39.1% | 27.9% | 33.1% |

Supplementary Table S4. Frequency of visiting cultural activities by educational level (Netherlands 2014, GLOBE: Dutch acronym of Health and Living Conditions of the Population of Eindhoven and surroundings).

|  | Never | Once per year | >Once per year |
| --- | --- | --- | --- |
| **Primary education** |  |  |  |
| Art museums | 90.7% | 7.3% | 2.0% |
| Historical museums | 88.8% | 10.5% | 0.7% |
| Opera | 96.0% | 2.7% | 1.3% |
| Classical concerts | 97.3% | 1.4% | 1.4% |
| Theatre | 82.8% | 13.3% | 4.0% |
| Architecture | 80.0% | 13.3% | 6.7% |
| **Lower secondary** |  |  |  |
| Art museums | 70.8% | 21.2% | 8.0% |
| Historical museums | 68.8% | 25.8% | 5.4% |
| Opera | 92.0% | 6.0% | 2.0% |
| Classical concerts | 86.4% | 8.8% | 4.9% |
| Theatre | 60.0% | 25.7% | 14.4% |
| Architecture | 51.8% | 26.4% | 21.9% |
| **Upper secondary** |  |  |  |
| Art museums | 62.6% | 27.0% | 10.4% |
| Historical museums | 59.6% | 34.2% | 6.2% |
| Opera | 88.9% | 8.9% | 2.2% |
| Classical concerts | 84.2% | 11.5% | 4.3% |
| Theatre | 46.6% | 36.5% | 17.0% |
| Architecture | 43.7% | 32.0% | 24.3% |
| **Tertiary** |  |  |  |
| Art museums | 37.2% | 39.2% | 23.6% |
| Historical museums | 41.7% | 47.2% | 11.1% |
| Opera | 79.4% | 16.4% | 4.2% |
| Classical concerts | 69.0% | 20.9% | 10.1% |
| Theatre | 29.5% | 46.3% | 24.2% |
| Architecture | 21.7% | 33.9% | 44.4% |

Supplementary Table S5. Frequency of visiting cultural activities by income group (Netherlands 2014, GLOBE: Dutch acronym of Health and Living Conditions of the Population of Eindhoven and surroundings).

|  | Never | Once per year | >Once per year |
| --- | --- | --- | --- |
| **<€1000/month** |  |  |  |
| Art museums | 71.7% | 19.1% | 9.3% |
| Historical museums | 70.8% | 23.4% | 5.8% |
| Opera | 91.4% | 6.3% | 2.3% |
| Classical concerts | 86.3% | 10.2% | 3.5% |
| Theatre | 70.1% | 22.7% | 7.3% |
| Architecture | 56.2% | 25.7% | 18.2% |
| **€1000 – €1500/month** |  |  |  |
| Art museums | 67.1% | 23.4% | 9.5% |
| Historical museums | 64.0% | 29.8% | 6.2% |
| Opera | 89.8% | 8.4% | 1.8% |
| Classical concerts | 86.1% | 10.5% | 3.4% |
| Theatre | 52.5% | 34.6% | 12.9% |
| Architecture | 49.2% | 28.5% | 22.3% |
| **€1500 – €2000/month** |  |  |  |
| Art museums | 57.5% | 29.4% | 13.1% |
| Historical museums | 55.4% | 36.3% | 8.3% |
| Opera | 87.9% | 10.2% | 2.0% |
| Classical concerts | 81.3% | 12.5% | 6.2% |
| Theatre | 42.0% | 39.7% | 18.3% |
| Architecture | 36.4% | 32.9% | 30.7% |
| **€2000 – €2500/month** |  |  |  |
| Art museums | 37.4% | 39.0% | 23.5% |
| Historical museums | 42.8% | 47.3% | 9.9% |
| Opera | 81.7% | 13.9% | 4.4% |
| Classical concerts | 70.1% | 19.7% | 10.2% |
| Theatre | 29.3% | 44.5% | 26.1% |
| Architecture | 23.0% | 33.7% | 43.3% |
| **>€2500/month** |  |  |  |
| Art museums | 31.5% | 42.6% | 25.8% |
| Historical museums | 38.4% | 51.2% | 10.4% |
| Opera | 74.3% | 20.3% | 5.4% |
| Classical concerts | 66.0% | 20.9% | 13.1% |
| Theatre | 27.2% | 45.6% | 27.2% |
| Architecture | 15.5% | 31.7% | 52.9% |

Supplementary Table S6. Spearman correlation coefficients between the cultural activities (Netherlands 2014, GLOBE: Dutch acronym of Health and Living Conditions of the Population of Eindhoven and surroundings).

|  | Art museums | Historical museums | Opera | Classical concerts | Theatre | Architecture |
| --- | --- | --- | --- | --- | --- | --- |
| Art museums | 1 |  |  |  |  |  |
| Historical museums | 0.64 | 1 |  |  |  |  |
| Opera | 0.36 | 0.28 | 1 |  |  |  |
| Classical concerts | 0.45 | 0.32 | 0.50 | 1 |  |  |
| Theatre | 0.35 | 0.30 | 0.31 | 0.26 | 1 |  |
| Architecture | 0.59 | 0.54 | 0.26 | 0.34 | 0.37 | 1 |

Note: all correlations (p<.001).

Supplementary Table S7. Factor loadings of the cultural activities (Netherlands 2014, GLOBE: Dutch acronym of Health and Living Conditions of the Population of Eindhoven and surroundings).

|  | Factor loadings |
| --- | --- |
| Art museums | 0.83 |
| Historical museums | 0.74 |
| Opera | 0.62 |
| Classical concerts | 0.68 |
| Theatre | 0.57 |
| Architecture | 0.75 |

Note: Eigenvalue of factor = 2.97.

Supplementary Figure S1. Cultural participation (in quintiles) by educational level and household equivalent income (Netherlands 2014, GLOBE: Dutch acronym of Health and Living Conditions of the Population of Eindhoven and surroundings).


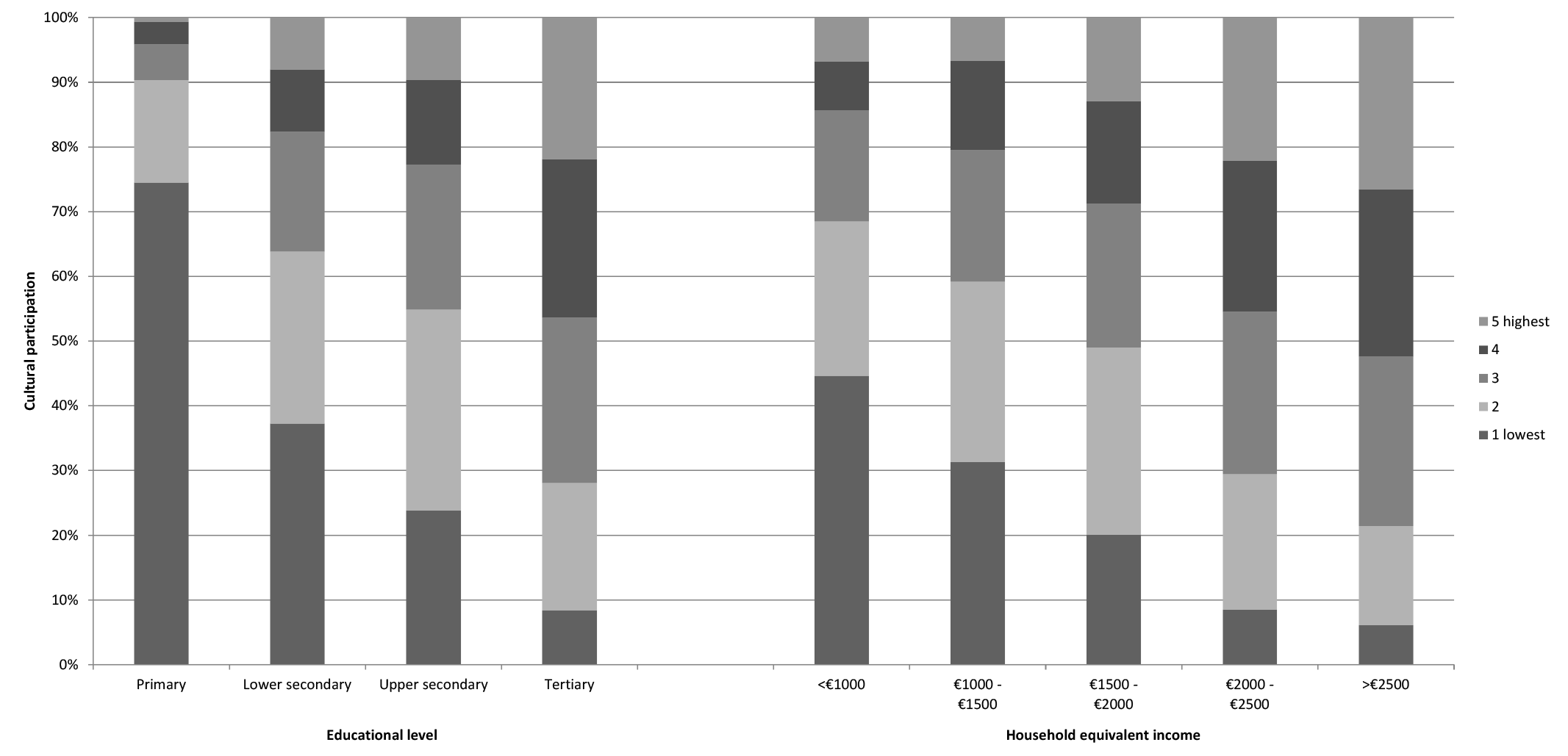


**Additional analyses**

Supplementary Table S8. Prevalence ratios of the association between cultural participation, educational level and income and ‘high status’ sports participation (golf, tennis, fencing, yoga, sailing, skiing, running and squash)^a^ (Netherlands 2014, GLOBE: Dutch acronym of Health and Living Conditions of the Population of Eindhoven and surroundings).

|  |  | **Crude model** | | | | **Education and income** | | | | **Adjusted model** | | | |
| --- | --- | --- | --- | --- | --- | --- | --- | --- | --- | --- | --- | --- | --- |
| **Variables** | **Categories** | **PR** | **95% CI** | | ***p*** | **PR** | **95% CI** | | ***p*** | **PR** | **95% CI** | | ***p*** |
| Cultural participation  (quintiles) | 1 lowest | 1 |  |  |  |  |  |  |  | 1 |  |  |  |
|  | 2 | 1.21 | 0.92 | 1.59 | 0.180 |  |  |  |  | 1.08 | 0.81 | 1.43 | 0.602 |
|  | 3 | 1.52 | 1.16 | 1.99 | 0.002 |  |  |  |  | 1.31 | 0.99 | 1.74 | 0.063 |
|  | 4 | 1.74 | 1.33 | 2.28 | <0.001 |  |  |  |  | 1.44 | 1.08 | 1.92 | 0.013 |
|  | 5 highest | 1.74 | 1.31 | 2.33 | <0.001 |  |  |  |  | 1.40 | 1.03 | 1.91 | 0.032 |
| Educational level | Primary |  |  |  |  | 1 |  |  |  | 1 |  |  |  |
|  | Lower secondary |  |  |  |  | 3.03 | 1.40 | 6.52 | 0.005 | 2.84 | 1.31 | 6.18 | 0.008 |
|  | Upper secondary |  |  |  |  | 3.18 | 1.49 | 6.78 | 0.003 | 2.88 | 1.33 | 6.23 | 0.007 |
|  | Tertiary |  |  |  |  | 4.20 | 1.97 | 8.95 | <0.001 | 3.54 | 1.62 | 7.71 | 0.002 |
| Household equivalent income | <€1000 |  |  |  |  | 1 |  |  |  | 1 |  |  |  |
|  | €1000 – €1500 |  |  |  |  | 1.07 | 0.78 | 1.49 | 0.665 | 1.05 | 0.75 | 1.46 | 0.782 |
|  | €1500 – €2000 |  |  |  |  | 1.09 | 0.79 | 1.50 | 0.608 | 1.04 | 0.75 | 1.44 | 0.815 |
|  | €2000 – €2500 |  |  |  |  | 1.22 | 0.89 | 1.67 | 0.212 | 1.15 | 0.84 | 1.57 | 0.399 |
|  | >€2500 |  |  |  |  | 1.19 | 0.84 | 1.69 | 0.317 | 1.11 | 0.78 | 1.57 | 0.556 |

^a^ Selection of ‘high status’ sports was based on Stempel (2005) and Scheerder et al. (2002).

All models were adjusted for confounders: sex, age, living together with a partner, country of birth, children living at home, employment status, social participation, mental health and self-rated health.

Supplementary Table S9. Prevalence ratios of the association between cultural participation, educational level and income and ‘non-high status’ sports participation ^a^ (Netherlands 2014, GLOBE: Dutch acronym of Health and Living Conditions of the Population of Eindhoven and surroundings).

|  |  | **Crude model** | | | | **Education and income** | | | | **Adjusted model** | | | |
| --- | --- | --- | --- | --- | --- | --- | --- | --- | --- | --- | --- | --- | --- |
| **Variables** | **Categories** | **PR** | **95% CI** | | ***p*** | **PR** | **95% CI** | | ***p*** | **PR** | **95% CI** | | ***p*** |
| Cultural participation  (quintiles) | 1 lowest | 1 |  |  |  |  |  |  |  | 1 |  |  |  |
|  | 2 | 1.14 | 0.94 | 1.37 | 0.181 |  |  |  |  | 1.08 | 0.90 | 1.31 | 0.409 |
|  | 3 | 1.16 | 0.95 | 1.40 | 0.139 |  |  |  |  | 1.10 | 0.91 | 1.34 | 0.327 |
|  | 4 | 1.14 | 0.92 | 1.40 | 0.222 |  |  |  |  | 1.09 | 0.88 | 1.35 | 0.448 |
|  | 5 highest | 1.09 | 0.87 | 1.36 | 0.438 |  |  |  |  | 1.05 | 0.83 | 1.32 | 0.699 |
| Educational level | Primary |  |  |  |  | 1 |  |  |  | 1 |  |  |  |
|  | Lower secondary |  |  |  |  | 1.39 | 0.95 | 2.02 | 0.086 | 1.35 | 0.93 | 1.97 | 0.114 |
|  | Upper secondary |  |  |  |  | 1.46 | 1.00 | 2.12 | 0.049 | 1.42 | 0.97 | 2.07 | 0.073 |
|  | Tertiary |  |  |  |  | 1.39 | 0.96 | 2.03 | 0.083 | 1.35 | 0.92 | 1.98 | 0.131 |
| Household equivalent income | <€1000 |  |  |  |  | 1 |  |  |  | 1 |  |  |  |
|  | €1000 – €1500 |  |  |  |  | 1.34 | 1.06 | 1.71 | 0.015 | 1.33 | 1.05 | 1.69 | 0.018 |
|  | €1500 – €2000 |  |  |  |  | 1.37 | 1.07 | 1.76 | 0.012 | 1.35 | 1.06 | 1.74 | 0.016 |
|  | €2000 – €2500 |  |  |  |  | 1.20 | 0.93 | 1.54 | 0.162 | 1.18 | 0.92 | 1.52 | 0.195 |
|  | >€2500 |  |  |  |  | 1.38 | 1.03 | 1.84 | 0.029 | 1.36 | 1.02 | 1.82 | 0.037 |

^a^ ‘ Non-high status’ sports include all sports except for those considered high status (see Appendix 3 Table 1).

All models were adjusted for confounders: sex, age, living together with a partner, country of birth, children living at home, employment status, social participation, mental health and self-rated health.

Supplementary Table S10. Linear regression model of the association between cultural participation, educational level and income and weekly fruit and vegetable consumption (per 100 grams/pieces of fruit) ^a^ (Netherlands 2014, GLOBE: Dutch acronym of Health and Living Conditions of the Population of Eindhoven and surroundings); N=2720.

|  |  | **Crude model** | | | |  | **Education and income** | | | |  | **Adjusted model** | | | |
| --- | --- | --- | --- | --- | --- | --- | --- | --- | --- | --- | --- | --- | --- | --- | --- |
| **Variables** | **Categories** | **β** | **95% CI** | | ***p*** |  | **β** | **95% CI** | | ***p*** |  | **β** | **95% CI** | | ***p*** |
| Cultural participation  (quintiles) | 1 lowest | 0 |  |  |  |  |  |  |  |  |  | 0 |  |  |  |
|  | 2 | 1.57 | 0.40 | 2.75 | 0.008 |  |  |  |  |  |  | 1.32 | 0.09 | 2.55 | 0.035 |
|  | 3 | 2.65 | 1.46 | 3.85 | <0.001 |  |  |  |  |  |  | 2.27 | 0.99 | 3.55 | 0.001 |
|  | 4 | 3.84 | 2.51 | 5.17 | <0.001 |  |  |  |  |  |  | 3.27 | 1.83 | 4.71 | <0.001 |
|  | 5 highest | 5.59 | 4.18 | 7.00 | <0.001 |  |  |  |  |  |  | 4.94 | 3.38 | 6.50 | <0.001 |
| Educational level | Primary |  |  |  |  |  | 0 |  |  |  |  | 0 |  |  |  |
|  | Lower secondary |  |  |  |  |  | 0.92 | -1.18 | 3.01 | 0.391 |  | 0.30 | -1.80 | 2.40 | 0.779 |
|  | Upper secondary |  |  |  |  |  | 1.30 | -0.85 | 3.44 | 0.235 |  | 0.20 | -1.97 | 2.38 | 0.856 |
|  | Tertiary |  |  |  |  |  | 3.49 | 1.32 | 5.66 | 0.002 |  | 1.59 | -0.66 | 3.84 | 0.166 |
| Household equivalent income | <€1000 |  |  |  |  |  | 0 |  |  |  |  | 0 |  |  |  |
|  | €1000 – €1500 |  |  |  |  |  | 1.10 | -0.39 | 2.59 | 0.146 |  | 0.83 | -0.65 | 2.31 | 0.272 |
|  | €1500 – €2000 |  |  |  |  |  | 0.97 | -0.67 | 2.60 | 0.246 |  | 0.48 | -1.16 | 2.13 | 0.564 |
|  | €2000 – €2500 |  |  |  |  |  | 1.15 | -0.50 | 2.80 | 0.171 |  | 0.31 | -1.34 | 1.96 | 0.715 |
|  | >€2500 |  |  |  |  |  | 0.87 | -1.09 | 2.82 | 0.385 |  | -0.04 | -1.99 | 1.91 | 0.967 |

^a^ Consumption of fruit and vegetables was calculated by adding up how much fruit and vegetables (per 100 grams) participants consumed in total in a typical week, where 100 grams was used as the equivalent of one piece of fruit.

All models were adjusted for confounders: sex, age, living together with a partner, country of birth, children living at home, employment status, social participation, mental health and self-rated health.

**Stratified analyses by gender and age groups**

Supplementary Table S11. Prevalence ratios of the association between cultural participation, educational level and income and the outcomes: men (Netherlands 2014, GLOBE: Dutch acronym of Health and Living Conditions of the Population of Eindhoven and surroundings).

|  | **Sports participation** | **Walking or cycling in leisure time** | **Recommended vegetable intake** | **Recommended fruit intake** |
| --- | --- | --- | --- | --- |
|  | **PR (95% CI)** | **PR (95% CI)** | **PR (95% CI)** | **PR (95% CI)** |
| **Cultural participation**  **(quintiles)** |  |  |  |  |
| 1 lowest | 1 | 1 | 1 | 1 |
| 2 | 1.09 (0.93-1.29) | 1.13 (0.97-1.31) | 0.99 (0.63-1.56) | 0.90 (0.64-1.27) |
| 3 | 1.12 (0.95-1.31) | 1.26 (1.09-1.45) | 1.44 (0.94-2.21) | 1.06 (0.75-1.49) |
| 4 | 1.12 (0.95-1.33) | 1.07 (0.91-1.26) | 1.09 (0.69-1.72) | 1.08 (0.74-1.57) |
| 5 highest | 1.17 (0.97-1.40) | 1.38 (1.19-1.60) | 1.35 (0.83-2.21) | 1.43 (0.98-2.10) |
| **Educational level** |  |  |  |  |
| Primary | 1 | 1 | 1 | 1 |
| Lower secondary | 1.23 (0.80-1.91) | 1.00 (0.81-1.25) | 1.10 (0.50-2.44) | 0.56 (0.36-0.86) |
| Upper secondary | 1.18 (0.76-1.83) | 0.95 (0.75-1.19) | 1.02 (0.46-2.24) | 0.44 (0.28-0.70) |
| Tertiary | 1.24 (0.80-1.92) | 0.87 (0.69-1.10) | 1.67 (0.75-3.70) | 0.45 (0.28-0.72) |
| **Household equivalent income** |  |  |  |  |
| <€1000/month | 1 | 1 | 1 | 1 |
| €1000 – €1500/month | 1.20 (0.94-1.54) | 0.97 (0.80-1.19) | 0.98 (0.58-1.65) | 1.25 (0.77-2.02) |
| €1500 – €2000/month | 1.36 (1.06-1.74) | 1.02 (0.84-1.24) | 0.90 (0.51-1.58) | 1.30 (0.78-2.17) |
| €2000 – €2500/month | 1.29 (1.01-1.65) | 1.01 (0.83-1.24) | 0.96 (0.55-1.66) | 1.39 (0.84-2.31) |
| >€2500/month | 1.42 (1.09-1.84) | 1.06 (0.85-1.32) | 0.91 (0.50-1.64) | 1.24 (0.71-2.19) |

All models were adjusted for confounders: sex, age, living together with a partner, country of birth, children living at home, employment status, social participation, mental health and self-rated health.

Supplementary Table S12. Prevalence ratios of the association between cultural participation, educational level and income and the outcomes: women (Netherlands 2014, GLOBE: Dutch acronym of Health and Living Conditions of the Population of Eindhoven and surroundings).

|  | **Sports participation** | **Walking or cycling in leisure time** | **Recommended vegetable intake** | **Recommended fruit intake** |
| --- | --- | --- | --- | --- |
|  | **PR (95% CI)** | **PR (95% CI)** | **PR (95% CI)** | **PR (95% CI)** |
| **Cultural participation**  **(quintiles)** |  |  |  |  |
| 1 lowest | 1 | 1 | 1 | 1 |
| 2 | 1.19 (0.99-1.42) | 1.16 (1.01-1.33) | 0.93 (0.65-1.34) | 1.16 (0.90-1.50) |
| 3 | 1.28 (1.07-1.54) | 1.19 (1.04-1.37) | 1.10 (0.78-1.55) | 1.25 (0.97-1.62) |
| 4 | 1.30 (1.08-1.56) | 1.37 (1.20-1.58) | 1.63 (1.14-2.32) | 1.43 (1.10-1.87) |
| 5 highest | 1.24 (1.01-1.50) | 1.34 (1.16-1.54) | 1.64 (1.14-2.36) | 1.67 (1.28-2.17) |
| **Educational level** |  |  |  |  |
| Primary | 1 | 1 | 1 | 1 |
| Lower secondary | 2.31 (1.41-3.76) | 1.02 (0.86-1.21) | 1.42 (0.72-2.79) | 0.90 (0.63-1.30) |
| Upper secondary | 2.54 (1.56-4.15) | 0.99 (0.82-1.19) | 1.35 (0.68-2.65) | 0.89 (0.61-1.31) |
| Tertiary | 2.77 (1.69-4.53) | 1.12 (0.93-1.36) | 1.63 (0.82-3.24) | 1.03 (0.70-1.51) |
| **Household equivalent income** |  |  |  |  |
| <€1000/month | 1 | 1 | 1 | 1 |
| €1000 – €1500/month | 1.16 (0.95-1.42) | 0.90 (0.79-1.04) | 1.05 (0.71-1.54) | 1.15 (0.86-1.54) |
| €1500 – €2000/month | 1.14 (0.93-1.40) | 0.99 (0.86-1.14) | 1.16 (0.78-1.73) | 1.28 (0.94-1.74) |
| €2000 – €2500/month | 1.10 (0.89-1.35) | 0.92 (0.79-1.06) | 1.10 (0.74-1.63) | 1.20 (0.88-1.64) |
| >€2500/month | 1.17 (0.92-1.48) | 0.96 (0.80-1.15) | 1.16 (0.74-1.81) | 1.26 (0.90-1.77) |

All models were adjusted for confounders: sex, age, living together with a partner, country of birth, children living at home, employment status, social participation, mental health and self-rated health.

Supplementary Table S13. Prevalence ratios of the association between cultural participation, educational level and income and the outcomes: ages 25-41 (Netherlands 2014, GLOBE: Dutch acronym of Health and Living Conditions of the Population of Eindhoven and surroundings).

|  | **Sports participation** | **Walking or cycling in leisure time** | **Recommended vegetable intake** | **Recommended fruit intake** |
| --- | --- | --- | --- | --- |
|  | **PR (95% CI)** | **PR (95% CI)** | **PR (95% CI)** | **PR (95% CI)** |
| **Cultural participation**  **(quintiles)** |  |  |  |  |
| 1 lowest | 1 | 1 | 1 | 1 |
| 2 | 1.06 (0.91-1.24) | 1.35 (1.05-1.72) | 1.15 (0.72-1.82) | 1.09 (0.74-1.61) |
| 3 | 1.05 (0.90-1.23) | 1.59 (1.24-2.03) | 1.58 (1.03-2.43) | 1.26 (0.85-1.86) |
| 4 | 1.12 (0.96-1.31) | 1.74 (1.34-2.25) | 1.86 (1.17-2.96) | 1.24 (0.82-1.89) |
| 5 highest | 0.98 (0.81-1.17) | 1.77 (1.33-2.35) | 1.85 (1.12-3.04) | 1.59 (1.02-2.47) |
| **Educational level** |  |  |  |  |
| Primary | 1 | 1 | 1 | 1 |
| Lower secondary | 2.55 (1.20-5.44) | 1.27 (0.75-2.15) | 1.68 (0.50-5.65) | 0.76 (0.27-2.13) |
| Upper secondary | 2.22 (1.05-4.69) | 1.05 (0.64-1.74) | 1.58 (0.51-4.93) | 0.92 (0.36-2.37) |
| Tertiary | 2.58 (1.22-5.46) | 0.98 (0.59-1.63) | 1.83 (0.59-5.65) | 1.02 (0.39-2.67) |
| **Household equivalent income** |  |  |  |  |
| <€1000/month | 1 | 1 | 1 | 1 |
| €1000 – €1500/month | 1.24 (1.03-1.50) | 0.98 (0.76-1.26) | 1.13 (0.69-1.83) | 1.06 (0.69-1.65) |
| €1500 – €2000/month | 1.23 (1.00-1.50) | 1.12 (0.87-1.46) | 1.24 (0.76-2.00) | 1.17 (0.75-1.83) |
| €2000 – €2500/month | 1.11 (0.91-1.35) | 1.04 (0.80-1.35) | 1.17 (0.72-1.90) | 1.14 (0.72-1.79) |
| >€2500/month | 1.21 (0.98-1.49) | 1.13 (0.84-1.52) | 1.36 (0.80-2.32) | 1.24 (0.76-2.05) |

All models were adjusted for confounders: sex, age, living together with a partner, country of birth, children living at home, employment status, social participation, mental health and self-rated health.

Supplementary Table S14. Prevalence ratios of the association between cultural participation, educational level and income and the outcomes: ages 42-58 (Netherlands 2014, GLOBE: Dutch acronym of Health and Living Conditions of the Population of Eindhoven and surroundings).

|  | **Sports participation** | **Walking or cycling in leisure time** | **Recommended vegetable intake** | **Recommended fruit intake** |
| --- | --- | --- | --- | --- |
|  | **PR (95% CI)** | **PR (95% CI)** | **PR (95% CI)** | **PR (95% CI)** |
| **Cultural participation**  **(quintiles)** |  |  |  |  |
| 1 lowest | 1 | 1 | 1 | 1 |
| 2 | 1.20 (0.93-1.55) | 1.32 (1.05-1.66) | 0.97 (0.57-1.65) | 1.18 (0.75-1.84) |
| 3 | 1.27 (0.99-1.64) | 1.27 (0.99-1.62) | 1.12 (0.64-1.94) | 1.27 (0.80-2.02) |
| 4 | 1.39 (1.08-1.78) | 1.29 (0.99-1.69) | 1.15 (0.65-2.01) | 1.54 (0.96-2.47) |
| 5 highest | 1.29 (0.98-1.71) | 1.71 (1.33-2.20) | 1.36 (0.75-2.44) | 1.88 (1.17-3.02) |
| **Educational level** |  |  |  |  |
| Primary | 1 | 1 | 1 | 1 |
| Lower secondary | 1.18 (0.61-2.26) | 1.26 (0.83-1.90) | 0.91 (0.37-2.26) | 0.74 (0.39-1.41) |
| Upper secondary | 1.52 (0.80-2.89) | 1.16 (0.77-1.76) | 0.84 (0.34-2.06) | 0.68 (0.35-1.31) |
| Tertiary | 1.67 (0.88-3.17) | 1.25 (0.82-1.90) | 1.20 (0.48-2.98) | 0.70 (0.36-1.37) |
| **Household equivalent income** |  |  |  |  |
| <€1000/month | 1 | 1 | 1 | 1 |
| €1000 – €1500/month | 1.25 (0.93-1.66) | 0.96 (0.77-1.19) | 1.12 (0.63-1.99) | 1.31 (0.80-2.15) |
| €1500 – €2000/month | 1.21 (0.91-1.61) | 0.95 (0.75-1.21) | 1.08 (0.57-2.04) | 1.24 (0.73-2.09) |
| €2000 – €2500/month | 1.13 (0.86-1.50) | 0.89 (0.70-1.14) | 1.09 (0.57-2.09) | 1.19 (0.70-2.02) |
| >€2500/month | 1.16 (0.84-1.60) | 1.01 (0.74-1.37) | 1.10 (0.53-2.26) | 1.32 (0.72-2.44) |

All models were adjusted for confounders: sex, age, living together with a partner, country of birth, children living at home, employment status, social participation, mental health and self-rated health.

Supplementary Table S15. Prevalence ratios of the association between cultural participation, educational level and income and the outcomes: ages 59-75 (Netherlands 2014, GLOBE: Dutch acronym of Health and Living Conditions of the Population of Eindhoven and surroundings).

|  | **Sports participation** | **Walking or cycling in leisure time** | **Recommended vegetable intake** | **Recommended fruit intake** |
| --- | --- | --- | --- | --- |
|  | **PR (95% CI)** | **PR (95% CI)** | **PR (95% CI)** | **PR (95% CI)** |
| **Cultural participation**  **(quintiles)** |  |  |  |  |
| 1 lowest | 1 | 1 | 1 | 1 |
| 2 | 1.13 (0.88-1.46) | 1.01 (0.91-1.11) | 0.80 (0.47-1.34) | 0.92 (0.67-1.25) |
| 3 | 1.29 (1.01-1.66) | 1.08 (0.99-1.18) | 1.15 (0.70-1.89) | 1.08 (0.79-1.47) |
| 4 | 1.03 (0.78-1.36) | 1.04 (0.95-1.15) | 1.28 (0.78-2.10) | 1.19 (0.87-1.63) |
| 5 highest | 1.29 (1.01-1.66) | 1.12 (1.02-1.22) | 1.46 (0.89-2.38) | 1.45 (1.08-1.97) |
| **Educational level** |  |  |  |  |
| Primary | 1 | 1 | 1 | 1 |
| Lower secondary | 1.65 (1.06-2.58) | 0.99 (0.88-1.12) | 1.53 (0.73-3.23) | 0.78 (0.56-1.09) |
| Upper secondary | 1.56 (0.97-2.50) | 1.01 (0.89-1.15) | 1.41 (0.63-3.17) | 0.69 (0.46-1.02) |
| Tertiary | 1.43 (0.90-2.29) | 0.99 (0.87-1.13) | 2.37 (1.09-5.14) | 0.74 (0.51-1.08) |
| **Household equivalent income** |  |  |  |  |
| <€1000/month | 1 | 1 | 1 | 1 |
| €1000 – €1500/month | 0.99 (0.68-1.46) | 0.93 (0.81-1.06) | 0.92 (0.54-1.56) | 1.19 (0.82-1.74) |
| €1500 – €2000/month | 1.22 (0.84-1.77) | 1.01 (0.88-1.15) | 1.09 (0.62-1.92) | 1.37 (0.92-2.05) |
| €2000 – €2500/month | 1.23 (0.84-1.80) | 1.00 (0.87-1.14) | 1.05 (0.61-1.81) | 1.34 (0.90-2.00) |
| >€2500/month | 1.43 (0.96-2.11) | 1.00 (0.86-1.15) | 0.77 (0.41-1.43) | 1.20 (0.77-1.86) |

All models were adjusted for confounders: sex, age, living together with a partner, country of birth, children living at home, employment status, social participation, mental health and self-rated health.

**Detailed information on the outcome variables**

***Sports participation***

Supplementary Table S16. Types of sports reported (only >10 listed) (Netherlands 2014, GLOBE: Dutch acronym of Health and Living Conditions of the Population of Eindhoven and surroundings).

| Sports | Reported number | Sports | Reported number |
| --- | --- | --- | --- |
| Exercise in fitness club | 613 | **Martial arts** | 46 |
| Running | 397 | **Dancing** | 41 |
| Tennis | 136 | **Squash** | 39 |
| Yoga | 114 | **Aqua aerobics** | 25 |
| Soccer | 95 | **Physiotherapy** | 25 |
| Bicycle racing | 95 | **Bootcamp** | 25 |
| Regular cycling | 77 | **Volleyball** | 24 |
| Walking | 73 | **Mind sports** | 16 |
| Mountain biking | 71 | **Aerobics** | 16 |
| Gymnastics | 57 | **Body pump** | 16 |
| Pilates | 55 | **Jogging** | 16 |
| Cardio | 51 | **Skating** | 15 |
| Golf | 49 | **Hockey** | 15 |
| Swimming | 47 | **Badminton** | 12 |

Supplementary Table S17. Number of sports reported (Netherlands 2014, GLOBE: Dutch acronym of Health and Living Conditions of the Population of Eindhoven and surroundings).

| Number of sports | Percentage |
| --- | --- |
| 0 | 43.5% |
| 1 | 33.9% |
| 2 | 16.0% |
| 3 | 5.3% |
| 4 | 1.3% |

***Walking and cycling in leisure time***

Supplementary Table S18. Number of days that participants walked or cycled in leisure time for 30 minutes or more (Netherlands 2014, GLOBE: Dutch acronym of Health and Living Conditions of the Population of Eindhoven and surroundings).

| Number of days | Walking | Cycling |
| --- | --- | --- |
| 0 | 66.2% | 42.0% |
| 1 | 9.0% | 19.2% |
| 2 | 7.3% | 13.8% |
| 3 | 3.5% | 6.9% |
| 4 | 2.2% | 4.6% |
| 5 | 3.2% | 5.8% |
| 6 | 1.7% | 2.9% |
| 7 | 6.8% | 4.8% |

***Fruit and vegetable consumption***

Supplementary Table S19. Percentage of respondents consuming the recommended amount of fruit (at least 2 pieces of fruit every day) and vegetables (at least 200 grams of vegetables every day) (Netherlands 2014, GLOBE: Dutch acronym of Health and Living Conditions of the Population of Eindhoven and surroundings).

| Recommended consumption | Fruit | Vegetables | Fruit & vegetables |
| --- | --- | --- | --- |
| Yes | 23.5% | 33.7% | 13.6% |
| No | 76.5% | 66.3% | 86.4% |

Supplementary Table S20. Quartiles of frequency of weekly fruit and vegetable consumption (per 100 grams) (Netherlands 2014, GLOBE: Dutch acronym of Health and Living Conditions of the Population of Eindhoven and surroundings).

| **Minimum** | **1^st^ quartile** | **Median** | **3^rd^ quartile** | **Maximum** |
| --- | --- | --- | --- | --- |
| 0.00 | 11.75 | 19.25 | 25.00 | 52.50 |
